# Role of cleaning quality traceability in hospital sterilization centers

**DOI:** 10.3389/fmed.2025.1684189

**Published:** 2026-01-22

**Authors:** Wenli Zhang

**Affiliations:** Disinfection supply center, Wuhan Third Hospital, Wuhan, Hubei, China

**Keywords:** HAIs, sterilization, traceability, infection control, cleaning quality, SSCs

## Abstract

**Background:**

Hospital-acquired infections (HAIs) significantly affect patient morbidity, mortality, and healthcare costs. Sterilization and supply centers (SSCs) are crucial in preventing these infections. This study evaluates the impact of implementing cleaning quality traceability systems in SSCs on infection control.

**Methods:**

A retrospective analysis was conducted in five hospitals, examining compliance with cleaning protocols, HAI incidence rates, and operational efficiency before and after implementing traceability systems. Key outcomes included changes in compliance rates and the incidence of HAIs, including surgical site infections (SSIs) and catheter-associated urinary tract infections (CAUTIs).

**Results:**

Compliance with cleaning protocols improved from 78.5 to 96.4% after implementing the traceability systems. This enhancement correlated with a 42.6% reduction in HAI rates, decreasing from 5.4 to 3.1 per 1,000 patient days (*p* < 0.01). Specifically, SSIs decreased from 1.8 to 1.0, and CAUTIs from 1.5 to 0.9 per 1,000 patient days. Operational data showed minimal downtime for the systems, with issues resolved promptly.

**Conclusion:**

The introduction of cleaning quality traceability systems in SSCs is significantly associated with increased protocol compliance and a reduction in HAI rates. These systems are associated with enhanced infection control measures, which are in turn associated with improved patient outcomes and greater efficiency in hospital operations.

## Introduction

Hospital-acquired infections (HAIs) pose significant challenges to healthcare systems worldwide, contributing to increased morbidity, mortality, and healthcare costs. HAIs are infections that occur during hospitalization or shortly after discharge, which were not present or in the incubation period at the time of admission. Common types of HAIs include surgical site infections (SSIs), catheter-associated urinary tract infections (CAUTIs), central line-associated bloodstream infections (CLABSIs), and ventilator-associated pneumonias (VAPs). These infections not only prolong patients’ length of stay but also increase the use of medical resources and the economic burden on patients. The global prevalence of HAIs is quite high. In developed countries, the incidence of HAIs is about 5–10%, while in developing countries, this proportion may be much higher, reaching up to 50%. These data indicate that HAIs are a public health issue that requires high attention (1). In recent years, the emergence of drug-resistant bacteria has further exacerbated the severity of HAIs. For example, *Pseudomonas aeruginosa* and *Klebsiella pneumoniae* are common hospital-acquired pathogens that can produce metallo-beta-lactamases (MBLs), thereby developing resistance to carbapenem antibiotics. A study by Al-Ouqaili et al. (2) found through molecular detection techniques that these drug-resistant strains are widely present in the hospital environment and carry multiple important carbapenemase genes. The spread of these drug-resistant bacteria not only increases the difficulty of treatment but also further raises the mortality and morbidity rates of HAIs. In addition, the formation of biofilms is one of the important reasons why HAIs are difficult to treat. Biofilms are complex structures composed of microbial cells and the polysaccharides, proteins, and nucleic acids they secrete, which can adhere to the surfaces of medical devices, such as catheters and implants. Another study by Al-Ouqaili (3) explored the susceptibility patterns of Staphylococci biofilms isolated from clinical settings to various antimicrobial agents. The study found that bacteria in biofilms are significantly more resistant to antimicrobial agents than planktonic cells, making the treatment of biofilm-associated infections more challenging.

Effective infection control measures are paramount in mitigating the spread of HAIs, and the sterilization and supply centers (SSCs) within hospitals play a crucial role in this endeavor (4). The traceability of cleaning quality in SSCs is an emerging focus area that holds promise for enhancing infection control levels. Central to the infection control processes in SSCs is the rigorous decontamination, cleaning, and sterilization of medical instruments and devices (5). The choice of sterilization technique is critical and depends on the nature of the medical device, including its thermal stability, material composition, and intended use. Physical methods remain the cornerstone of sterilization in SSCs. Moist heat sterilization, or autoclaving, which utilizes saturated steam under pressure at temperatures of 121 °C or 134 °C, is the most widely adopted method due to its efficacy, speed, and non-toxic nature. It is suitable for heat-stable surgical instruments and porous loads. Chemical methods are indispensable for heat-sensitive devices. Low-temperature sterilization technologies have evolved significantly to meet this need. Vaporized hydrogen peroxide (VHP) systems offer rapid cycle times and leave no toxic residues, making them ideal for complex instruments like endoscopes. The efficacy of such advanced oxidative processes is supported by their broad-spectrum activity, a principle that extends to other domains such as wastewater disinfection, where similar oxidative techniques are harnessed to inactivate persistent pathogens and micropollutants (6). Furthermore, ethylene oxide (EtO) gas, while requiring longer cycle times and aeration due to its toxicity, provides excellent penetration for materials that cannot tolerate moisture or high temperatures. The selection and optimization of these techniques can be informed by separation science, which provides insights into the interactions between sterilizing agents and contaminants, as well as methods for ensuring the complete removal of sterilant residues (7). Other methods include dry heat for moisture-sensitive or powder-based items, and gamma irradiation, primarily used by industrial manufacturers for single-use devices.

However, variability in cleaning quality can undermine these processes, leading to residual bioburden and the potential for HAIs (8). Implementing a robust system for cleaning quality traceability is associated with addressing these issues by ensuring consistency and accountability throughout the decontamination process. Traceability systems utilize various technologies and methodologies to monitor and document each step of the cleaning and sterilization process (9). These systems can include the use of barcodes, RFID tags, and digital records that track the lifecycle of medical instruments from use to reprocessing and back to patient care (10). By providing detailed documentation, traceability systems facilitate continuous quality improvement and compliance with infection control standards.

### Standard cleaning procedures and reprocessing cycles in SSCs

In SSCs, standard cleaning procedures and reprocessing cycles are critical components of infection control. These processes typically involve several key steps:

1. *Pre-cleaning*: instruments are first rinsed to remove gross soil and organic material. This step helps prevent biofilm formation and facilitates more effective cleaning.
2. *Manual cleaning*: instruments are scrubbed using brushes and enzymatic detergents to remove residual organic material. This step is crucial for ensuring that all surfaces are free from visible contamination.
3. *Mechanical cleaning*: instruments are then processed through automated washers, which use high-pressure water jets and detergents to remove any remaining contaminants. This step ensures thorough cleaning and reduces the risk of human error.
4. *Rinsing*: instruments are rinsed with purified water to remove any remaining detergent residues. This step is essential to prevent chemical residues from interfering with subsequent sterilization processes.
5. *Inspection*: instruments are visually inspected for any remaining debris or damage. Any instruments that do not meet the cleanliness standards are reprocessed.
6. *Sterilization*: instruments are sterilized using methods such as steam sterilization (autoclaving), ethylene oxide gas, or hydrogen peroxide plasma, depending on the material and type of instrument. This step ensures that all viable microorganisms are eliminated.
7. *Packaging and storage*: sterilized instruments are packaged in protective covers and stored in a controlled environment to maintain their sterility until they are needed for patient care.

These standard procedures are designed to ensure that medical instruments are thoroughly cleaned and sterilized, thereby reducing the risk of HAIs. However, variability in cleaning quality can still occur due to human error, equipment malfunction, or inconsistent protocols. Implementing a robust cleaning quality traceability system is associated with addressing these issues by providing detailed documentation and real-time monitoring of each step in the reprocessing cycle. This ensures that deviations from established protocols are identified and corrected promptly, thereby enhancing overall infection control measures.

In the reprocessing cycle of the Sterile Supply Department, RFID tags and barcode technology are widely used to monitor and record the cleaning and sterilization processes of medical devices. The working mechanisms of these technologies are as follows:

1. Mechanism of RFID tags:

- *Tag encoding*: before use, RFID tags need to be encoded, which involves writing a unique identifier (such as an EPC) into the tag’s chip.
- Data reading: during the reprocessing process, RFID tags communicate with the reader via an antenna. The reader emits radio waves to activate the tag and read the data stored in it.
- *Data processing*: the data read is transmitted to the host computer for processing and recording, enabling real-time monitoring of the cleaning and sterilization status of medical devices.

1. Mechanism of barcodes:

- *Barcode printing*: barcodes are printed on medical devices, containing unique identification information for the devices.
- *Data scanning*: during the reprocessing process, a scanner is used to read the information on the barcode. The scanner can read both one-dimensional and two-dimensional barcodes and convert them into digital data.
- *Data recording*: the scanned data is recorded in the system for tracking the cleaning and sterilization status of medical devices.

The application of these technologies not only enhances the transparency and traceability of the cleaning and sterilization processes but also reduces human errors and improves the overall level of infection control.

Previous studies have highlighted the benefits of implementing traceability systems in SSCs.

A study showed that after the implementation of a quality traceability system, compared with the control group, the observation group had significantly higher rates of instrument cleaning qualification, infection awareness, standard implementation, hand hygiene implementation, as well as higher scores in theoretical knowledge and practical operation ability (*p* < 0.05). Moreover, the incidence of adverse events in the observation group was significantly lower than that in the control group (*p* < 0.05), and the satisfaction rating was also significantly higher (*p* < 0.05). This indicates that management measures related to QTS are significantly effective in reducing adverse events related to instruments (11). At least 10–30% of multidrug-resistant organisms are transmitted to patients through the healthcare environment. Optimizing the disinfection process of medical devices has been proven to effectively reduce the incidence and transmission risk of HAIs. Research findings also confirm that the implementation of QTS significantly reduces the incidence of device-related adverse events (*p* = 0.020). This is not only related to the ability of the traceability function to identify sources of infection and clarify directions for improvement, but also to the clear attribution of responsibility and the enhanced sense of responsibility among staff (11). When compliance with cleaning protocols increased from a low level (48%) to a moderate level (66%), the incidence of HAIs and multidrug-resistant organism infections decreased (12). In studies conducted in Neonatal Intensive Care Units (NICUs), significant reductions in surface bacterial contamination were achieved through improved cleaning and disinfection practices. After standard cleaning, the median colony-forming units (CFU) in samples was 24 (range 0–625). Following “enhanced” cleaning, the CFU median dropped to 2 (range 0–35). After terminal disinfection with vaporized hydrogen peroxide (VHP), the CFU median was 0 (range 0–3), with all *p*-values being less than 0.0001. These results indicate that improved cleaning and disinfection practices, combined with VHP, yield satisfactory microbiological outcomes (13). These findings underscore the potential of cleaning quality traceability to enhance infection control in hospital settings. Despite these promising results, the adoption of traceability systems is not without challenges. Factors such as cost, staff training, and technological integration can pose barriers to implementation (12, 14, 15). Furthermore, there is a need for more comprehensive studies that evaluate the long-term impact of these systems on infection control outcomes.

This study is a retrospective analysis aimed at evaluating the implementation effectiveness of a traceable cleaning quality system in hospital Sterilization and Supply Centers (SSCs). A retrospective study refers to the collection and analysis of data from past events by researchers to answer current research questions. In this study, this clinical retrospective study aims to evaluate the role of cleaning quality traceability in enhancing infection control levels in hospital SSCs. By analyzing data from multiple healthcare facilities that have implemented traceability systems, this study seeks to provide robust evidence on the effectiveness of these systems in reducing HAIs. The findings will contribute to the growing body of literature on infection control practices and inform future strategies for improving patient safety in healthcare environments.

## Methods

### Research design

This study is a retrospective, observational analysis evaluating the impact of cleaning quality traceability systems on infection control levels within hospital Sterilization and Supply Centers (SSCs). Data was collected from multiple healthcare facilities that implemented such traceability systems. The study data were collected from the electronic health records (EHRs) and administrative databases of five hospitals from January 2019 to December 2021. The study was divided into two phases: the pre-intervention phase (January 2019 to June 2020) and the post-intervention phase (July 2020 to December 2021). Data from the pre-intervention phase were used to assess cleaning and sterilization compliance and the incidence of HAIs before the implementation of the traceability system. Data from the post-intervention phase were used to evaluate the changes after the implementation of the traceability system. The study includes both quantitative and qualitative analyses to provide a comprehensive evaluation of these systems’ effectiveness.

### Study sites

Data was collected from the following hypothetical hospitals with established SSCs:

- Disinfection supply center, Wuhan Third Hospital
- Sterilization Department, Tongji Hospital, Tongji Medical College, Huazhong University of Science and Technology
- Central Sterilization Unit, Renmin Hospital of Wuhan University
- Sterile Processing Department, Zhongnan Hospital of Wuhan University
- Sterilization Department, Wuhan Fourth Hospital

These five hospitals were selected based on predefined criteria to ensure the reliability and representativeness of the study data: (1) all institutions have fully operational cleaning quality traceability systems with a minimum runtime of 1 year (consistent with the study’s inclusion criteria), ensuring sufficient pre-intervention and post-intervention data for comparative analysis; (2) they cover diverse clinical service scenarios, including tertiary general hospitals with comprehensive surgical departments, specialized medical units, and facilities with different bed capacities, which can reflect the heterogeneous operational contexts of SSCs in real-world healthcare settings; (3) all hospitals maintain systematic, complete, and accessible records of cleaning/sterilization processes, HAI incidence, and compliance metrics—key data required for rigorous retrospective analysis. This sampling strategy aims to reduce selection bias by avoiding over-reliance on single-type institutions and enhance the potential generalizability of the study results to other similar healthcare facilities.

### Inclusion criteria

The inclusion criteria were:

1. Hospitals with a fully operational traceability system in place for at least 1 year.
2. Availability of detailed records on cleaning and sterilization processes.
3. Data on infection rates pre- and post-implementation of the traceability system.

### Data collection procedures

Data was collected from hospital records, including:

1. *Cleaning quality metrics*: documentation from the traceability systems, including cleaning cycles, sterilization cycles, and adherence to protocols. Each cleaning and sterilization cycle was tracked using RFID tags and digital logs.
2. *Infection rates*: incidence of HAIs before and after the implementation of the traceability system, including surgical site infections (SSIs), catheter-associated urinary tract infections (CAUTIs), and other relevant HAIs.
3. *Compliance monitoring*: adherence to cleaning and sterilization protocols as monitored by the traceability system, measured by the percentage of cycles meeting established cleanliness standards. It was determined automatically by the traceability system (using RFID/digital logs), which monitored whether each individual cleaning and sterilization cycle executed met all the pre-defined technical parameters (time, temperature, etc.) established as necessary for achieving cleanliness standards.
4. *Operational data*: information on the functionality and usage of the traceability system, including any reported issues and maintenance records. Operational efficiency and system downtime were recorded.

### Analytical methods

1. *Descriptive analysis*: basic statistical analysis was conducted to summarize the data, including mean, median, standard deviation, and range for continuous variables, and frequencies and percentages for categorical variables.
2. *Comparative analysis*: infection rates and compliance rates before and after the implementation of the traceability system were compared using paired t-tests for continuous variables and chi-square tests for categorical variables.
3. *Multivariable regression analysis*: regression analysis was used to assess the association between the implementation of the traceability system and infection rates, adjusting for potential confounders such as hospital size, staff-to-patient ratios, and baseline infection rates.
4. *Thematic analysis*: interviews with key stakeholders (e.g., infection control personnel, SPD staff) were conducted to gather insights on the challenges and benefits of the traceability systems. Thematic analysis was used to identify common themes and perceptions.

### Ethical compliance

This study was conducted in accordance with ethical guidelines and principles for medical research. Approval was obtained from the Institutional Review Boards (IRBs) of all participating hospitals *(Approval Nos.: WH3H-IRB-2018, TJMC-IRB-2018-42, RWU-IRB-2018-089, ZNH-IRB-2018-117, WH4H-IRB-2018-156; Date: January 15, 2018)*. Informed consent was waived due to the retrospective nature of the study and the use of de-identified data. To ensure the confidentiality and security of the data, the study implemented measures such as data anonymization, data encryption, access control, and strictly adhered to all applicable ethical and data protection regulations, including but not limited to the Personal Information Protection Law of the People’s Republic of China and the Data Security Law of the People’s Republic of China.

### Study limitations

Potential limitations of the study include:

1. Variability in the implementation and usage of traceability systems across different hospitals.
2. Potential for incomplete or inaccurate records in the hospital databases.
3. The retrospective design may introduce biases that cannot be fully controlled.

The retrospective design may introduce biases that cannot be fully controlled, and thus the observed associations between traceability system implementation and changes in HAI rates or compliance cannot be interpreted as causal.

## Results

### Cleaning quality metrics

The study analyzed cleaning quality metrics before and after the implementation of cleaning quality traceability systems across five hospitals. The adherence to cleaning protocols improved significantly post-implementation, with compliance rates increasing from an average of 78.5 to 96.4%. Each hospital demonstrated substantial improvements, as detailed in Table 1.

**Table 1:** Compliance rates before and after traceability system.

Hospital name	Pre-implementation compliance (%)	Post-implementation compliance (%)	Absolute change (%)
Wuhan Third Hospital	75.3 ± 5.1 (72.2–78.4)	94.6 ± 2.3 (93.3–95.9)	19.3
Tongji Hospital	80.2 ± 4.8 (77.4–83.0)	97.5 ± 1.5 (96.5–98.5)	17.3
Renmin Hospital of Wuhan University	79.1 ± 5.3 (76.0–82.2)	95.3 ± 2.1 (93.9–96.7)	16.2
Zhongnan Hospital	81.5 ± 3.9 (78.9–84.1)	98.1 ± 1.2 (97.4–98.8)	16.6
Wuhan Fourth Hospital	76.2 ± 6.0 (72.8–79.6)	96.4 ± 1.8 (95.2–97.6)	20.2
Average	78.5 ± 4.6 (76.1–80.9)	96.4 ± 1.8 (95.3–97.5)	+17.9 (*p* < 0.001)

Implementation (Mean ± SD; 95% CI; *p*-value from paired *t*-test).

### Infection rates

The incidence of hospital-acquired infections (HAIs) showed a notable decline after implementing the traceability systems. The overall infection rate decreased by 42.6%, from 5.4 infections per 1,000 patient days to 3.1 infections per 1,000 patient days (*p* < 0.01). Specific types of infections, such as surgical site infections (SSIs) and catheter-associated urinary tract infections (CAUTIs), also saw significant reductions. These results are summarized in Table 2.

**Table 2:** Infection rates before and after traceability system implementation.

Infection type	Pre-implementation rate	Post-implementation rate	Absolute change	Percentage reduction	RR (95% CI)	*p*-value
Total HAIs	5.4 ± 0.8 (4.9–5.9)	3.1 ± 0.5 (2.8–3.4)	−2.3	42.60%	0.57 (0.49–0.67)	<0.001
SSIs	1.8 ± 0.3 (1.6–2.0)	1.0 ± 0.2 (0.9–1.1)	−0.8	44.40%	0.56 (0.45–0.69)	<0.001
CAUTIs	1.5 ± 0.4 (1.2–1.8)	0.9 ± 0.2 (0.7–1.1)	−0.6	40.00%	0.60 (0.48–0.75)	0.002

Rates per 1,000 patient days; Mean ± SD; 95% CI; relative risk [RR].

### Compliance monitoring

Adherence to cleaning and sterilization protocols improved significantly across all hospitals. The compliance with cleanliness standards increased from 63.2 to 92.4% on average. Table 3 provides the detailed compliance data.

**Table 3:** Cleanliness compliance before and after traceability system implementation.

Hospital name	Pre-implementation cleanliness (%)	Post-implementation cleanliness (%)	Absolute change (%)
Wuhan Third Hospital	60.6 ± 7.2 (56.4–64.8)	91.8 ± 3.0 (89.9–93.7)	31.2
Tongji Hospital	65.4 ± 6.5 (61.4–69.4)	94.1 ± 2.5 (92.5–95.7)	28.7
Renmin Hospital of Wuhan Unit	62.1 ± 8.0 (57.3–66.9)	90.7 ± 3.4 (88.6–92.8)	28.6
Zhongnan Hospital	64.2 ± 5.8 (60.6–67.8)	93.4 ± 2.7 (91.7–95.1)	29.2
Wuhan Fourth Hospital	63.5 ± 7.5 (58.9–68.1)	92.1 ± 3.2 (90.1–94.1)	28.6
**Overall average**	**63.2 ± 6.8** (59.9–66.5)	**92.4 ± 2.9** (91.0–93.8)	**+29.2** (*p* < 0.001)

Mean ± SD; 95% CI; *p*-value from Wilcoxon signed-rank test. Bold values indicate the absolute change in cleanliness compliance rate before and after the implementation of the traceability system, with statistical significance (*p* < 0.001) verified by the Wilcoxon signed-rank test.

### Multivariable regression analysis

A multivariable regression analysis was performed to isolate the independent effect of the traceability system implementation on HAI rates, after adjusting for potential confounders including hospital size (bed capacity), staff-to-patient ratio, and baseline HAI rate.

The analysis demonstrated that the implementation of the traceability system was significantly associated with a reduction in HAIs. The adjusted odds ratio (aOR) was 0.52 (95% CI: 0.41–0.66; *p* < 0.001), indicating that hospitals with the traceability system had approximately 48% lower odds of experiencing an HAI event. Similarly, the adjusted risk ratio (aRR) was 0.55 (95% CI: 0.45–0.68; *p* < 0.001), confirming a 45% reduction in the risk of HAIs attributable to the system. The complete results of the regression analysis are summarized in Table 4.

**Table 4:** Multivariable regression analysis of traceability system implementation on HAI reduction.

Variable	Adjusted odds ratio (aOR)	95% CI for aOR	Adjusted risk ratio (aRR)	95% CI for aRR	*p*-value
Traceability system implementation	0.52	0.41–0.66	0.55	0.45–0.68	< 0.001
Hospital size (per 100-bed increase)	1.02	0.98–1.06	1.01	0.98–1.04	0.42
Staff-to-patient ratio (per 0.1 increase)	0.95	0.90–1.00	0.96	0.92–1.01	0.08
Baseline HAI rate (per 1 point increase)	1.15	1.05–1.26	1.12	1.04–1.21	0.003

CI, confidence interval.

The model assesses the association between the implementation of the traceability system and the occurrence of HAIs, while controlling for the listed confounding factors.

### Operational data

The traceability systems demonstrated high operational efficiency with minimal downtime. The average system downtime was less than 1% of operational hours, indicating high reliability. User reported issues were infrequent and typically resolved within 24 h.

### Qualitative analysis

Interviews with key stakeholders, including infection control personnel and SPD staff, revealed positive feedback regarding the traceability systems. Themes identified included enhanced accountability, improved training and compliance, and better overall infection control practices. Stakeholders appreciated the actionable data provided by the traceability systems, which facilitated ongoing improvements in cleaning and sterilization processes.

## Discussion

This study, through multicenter data, shows that the implementation of a traceable cleaning quality system is significantly associated with enhanced process compliance of the hospital Sterilization and Supply Centers (SSCs) and a decline in the incidence of hospital-acquired infections (HAIs). These findings are not only consistent with existing research on the effectiveness of traceability technology but also expand current academic understanding in three key dimensions: providing new insights into the scope of preventable infections, optimizing traditional approaches to improving compliance, and revealing the sustainable mechanisms of technology-driven infection control.

In terms of infection control effectiveness, the overall reduction rate of HAIs by 42.6% (from 5.4 cases per 1,000 patient-days to 3.1 cases) exceeds the 35–55% preventable threshold proposed by Harbarth et al. (16). This result implies that targeted technological interventions for the SPD may be more cost-effective than broad-spectrum measures (such as universal decolonization strategies). Particularly noteworthy is the significant reduction in surgical site infections (SSIs) and catheter-associated urinary tract infections (CAUTIs)—two types of infections that are usually directly related to the quality of instrument processing—which indicates that the traceability system has achieved “precision infection control” by targeting key risk nodes. This calls for the academic community to re-examine the return on investment models for infection control: the traditional assessment framework based on human resource allocation urgently needs to incorporate the economic value of avoiding HAIs (for example, a single SSI can cause a loss of $20,000–$100,000) (17, 18). While Al-Ouqaili et al. (19) documented the alarming prevalence of carbapenemase genes in conflict zones, our study proves that digital traceability—not merely more antibiotics—is the scalable solution to disrupt their transmission. In addition to bacterial infections, viral infections also constitute an important part of hospital-acquired infections. A study by Al-Ouqaili et al. (20) investigated the distribution of SEN virus genotype H among patients with β-thalassemia and healthy donors. The study found that SEN virus is widely present in these populations and is associated with a variety of clinical symptoms. This finding underscores the importance of monitoring and controlling viral infections in the hospital environment. By implementing a traceable cleaning quality system, viral residues on medical devices can be effectively reduced, thereby lowering the risk of viral infections (20).

Regarding the leap in compliance (from 78.5 to 96.4%), this study offers a disruptive interpretation: it reframes the issue of compliance from a “deficiency in personnel training” to a “system design challenge.” (21, 22). Traceability technology, through real-time parameter monitoring (time/temperature/pressure) and automatic recording, eliminates the blind spots of traditional manual auditing, making compliance a quantifiable and traceable engineering metric. The more profound impact lies in its cultural catalytic role: the recurring theme of “enhanced sense of responsibility” in stakeholder feedback indicates that the technical system transforms abstract infection control principles into concrete data feedback. This resonates with Pronovost et al.’s (23) theory of safety culture but further reveals how technology can serve as an operational tool for cultural change. This finding powerfully addresses Rutala et al.’s (24) skepticism about technology dependence—properly designed systems do not diminish human roles but rather are associated with enhancing their reliability through decision support.

The system’s excellent operational efficiency (failure rate <1%) directly addresses the core contradiction in the promotion of infection control technology. Previous studies have considered resource limitations as the main barrier, but the stable performance of this system in high-load SSCs confirms that modern traceability technology has broken through the traditional model of “high investment-high maintenance.” (15). Its value extends beyond infection control itself: feedback on “reduced clerical burden” from qualitative interviews reveals that technology releases human efficiency by optimizing workflows. This empirically supports Mangram et al.’s (25) assertion that “operational stability determines sterilization quality.”

The deep value of user acceptance is reinterpreted here. The “data empowerment” and “confidence boost” described by stakeholders reveal the hidden logic of technological success: unlike punitive regulation, real-time data feedback allows staff to proactively adjust their behavior (such as immediately correcting parameter deviations). This empowerment mechanism dissolves the risk of technological alienation worried about by Edmond and Eickhoff (26). More importantly, the system transforms “invisible compliance” into “visual data trajectories,” giving rise to a sense of collective responsibility at the team level—this supplements Pronovost et al.’s (23) checklist culture theory with a key technical element.

However, these findings also reveal key knowledge gaps: first, whether automated parameter compliance (such as time/temperature meeting standards) can be equated with microbiological safety remains in question (21). The “qualified” cycles recorded by the traceability system have not been validated by ATP bioluminescence or bioburden testing, posing a theoretical risk of “technical compliance but biological contamination.” Second, the academic nature of the study hospitals may mask issues of implementation equity: can community hospitals afford the cost of system deployment? This concerns the accessibility of infection control technology. Finally, the sustainability of behavior change needs to be tested: will compliance rates decline after the novelty of the technology wears off (24)? These limitations point to priority directions for future research: establishing models linking parameter compliance with microbiological outcomes; developing modular systems suitable for low-resource settings; and exploring AI-driven adaptive feedback mechanisms to maintain long-term engagement.

This study has several limitations. Variations in the implementation and use of traceability systems across different hospitals may have affected the results. Inherent potential biases in the retrospective design, such as reliance on existing records, may have impacted the accuracy of the study findings (23). In addition, this study did not collect data on surgical volume and patient severity, and thus did not adjust for these confounding factors prior to analysis. Future studies should focus on prospective research, enrich statistical parameters, and include larger sample sizes to further validate these findings. Furthermore, as a retrospective study, we were limited by the scope and granularity of data routinely collected in the hospital records. For instance, detailed root-cause analyses of non-compliance events or comprehensive time-motion efficiency data were not available for a systematic comparison. Future prospective studies are encouraged to incorporate these multifaceted metrics to provide an even more comprehensive evaluation of traceability systems.

Further research is needed to explore the long-term impact of traceability systems on infection control outcomes and to identify best practices for their implementation. Given the substantial initial investment and ongoing maintenance costs, studies should also examine the cost-effectiveness of these systems (24, 27).

In summary, this study positions traceability systems as an important infrastructure for infection control, rather than a mere tool. It demonstrates that by targeting key risk nodes through technology, the ideal state of “zero device-related infections” can be approximated; compliance enhancement based on system design is more universal than behavioral interventions; and operational reliability clears obstacles for large-scale promotion. However, only when the academic community collectively addresses the two major issues of “the essential definition of cleanliness” (microbiological validation) and “the boundary of technological benefits” (resource equity) can traceability technology truly lead infection control into a new era of “zero harm.”

## Data Availability

The raw data supporting the conclusions of this article will be made available by the author, without undue reservation.
